# Guaiacol

**Published:** 1895-09

**Authors:** 


### Guaiacol.

Among the thereapeutic possibilities of guaiacol, its use upon mucous membranes is important. As an antiseptic it is almost equal to carbolic acid, while pure guaiacol is devoid of the caustic properties of carbolic acid. It can, therefore, be applied undiluted to the mucous membrane of the throat and nose, if not used in excessive quantity. It stings, but the unpleasant sensation soon passes off. It is often curative in tonsillitis; it is a prophylactic of apparent power against diphtheria; it is a useful agent in cases of tuberculous ulceration of the throat; and is one of the best of topical applications in ozena and other nasal affections. In the case of children and others to whom the undiluted drug might possibly prove too severe, it may be diluted one-half or two-thirds with glycerine or any bland menstruum (olive oil, cotton-seed oil, cocoanut oil, liquid petrolatum). To get good results from guaiacol, whether used internally or topically, one must have a pure preparation. Some of the specimens found in the shops are contaminated with impurities possessing more or less caustic quality. —*Philadelphia Polyclinic.*

